# Explaining large mitochondrial sequence differences within a population sample

**DOI:** 10.1098/rsos.170730

**Published:** 2017-11-29

**Authors:** Mary Morgan-Richards, Mariana Bulgarella, Louisa Sivyer, Edwina J. Dowle, Marie Hale, Natasha E. McKean, Steven A. Trewick

**Affiliations:** 1 Ecology, Massey University, Private Bag 11 222, Palmerston North, New Zealand; 2 Department of Integrative Biology, University of Colorado, 1151 Arapahoe, SI 2071, Denver, CO 80204, USA; 3 School of Biological Sciences, University of Canterbury, Christchurch, New Zealand

**Keywords:** constraining selection, DNA barcoding, genetic drift, *Hemideina*, mtDNA divergence, population size

## Abstract

Mitochondrial DNA sequence is frequently used to infer species' boundaries, as divergence is relatively rapid when populations are reproductively isolated. However, the shared history of a non-recombining gene naturally leads to correlation of pairwise differences, resulting in mtDNA clusters that might be mistaken for evidence of multiple species. There are four distinct processes that can explain high levels of mtDNA sequence difference within a single sample. Here, we examine one case in detail as an exemplar to distinguish among competing hypotheses. Within our sample of tree wētā (*Hemideina crassidens*; Orthoptera), we found multiple mtDNA haplotypes for a protein-coding region (*cytb*/*ND1*) that differed by a maximum of 7.9%. From sequencing the whole mitochondrial genome of two representative individuals, we found evidence of constraining selection. Heterozygotes were as common as expected under random mating at five nuclear loci. Morphological traits and nuclear markers did not resolve the mtDNA groupings of individuals. We concluded that the large differences found among our sample of mtDNA sequences were simply owing to a large population size over an extended period of time allowing an equilibrium between mutation and drift to retain a great deal of genetic diversity within a single species.

## 1. Introduction

High levels of mitochondrial sequence divergence can be misconstrued as indicating the presence of more than one species if DNA barcoding approaches are applied naively. Although the acquisition of mitochondrial sequence data has been promoted as a tool for identification of specimens [1,2], the application of mtDNA data frequently raises questions rather than discriminating among hypotheses [3]. However, the capacity for assigning specimens to previously identified, classified and sequenced maternal lineages is valuable for many aspects of biology which require this kind of identification. Issues relating to biosecurity, forensics, conservation and animal diet where extensive reference collections have been made with the necessary investment in mtDNA or cpDNA sequencing benefit from this approach, e.g. [4–8]. Unfortunately, 15 years since its introduction the fundamental flaws of DNA barcoding as an approach for taxonomic discovery [9–11] are still often ignored. MtDNA ‘barcode’ sequence variation above approximately 3% coupled with spatial structure or sympatry tends to lead to inferences of morphologically cryptic taxonomic diversity, even though theory provides other explanations. The presence within a single interbreeding population of multiple mtDNA lineages or haplogroups that differ by more than 3% sequence divergence is, for example, likely if populations have been large for many generations. Here, we demonstrate how hypotheses that explain this pattern can be distinguished by using a New Zealand orthopteran as a case study.

Four biological processes might lead to high genetic diversity at a locus within a single population sample. In a stable population, neutral genetic variants arise at a predictable rate and are lost stochastically (drift) at a rate determined by population size [12,13]. In large populations, the presence of many individuals increases the potential for new mutations each generation and the rate of allele loss via drift is relatively slow. Typically, therefore, invertebrate populations with relatively small individuals and large population size contain much more genetic diversity than large vertebrate species with their small effective populations, for example, compare mammalian and avian host with insect parasite diversity levels, e.g. [14–16]. High genetic diversity within a sample might therefore be owing to persistence of a large population at equilibrium between mutation and drift [13]. Large constant population sizes result in a distribution of pairwise differences that deviate from the expected exponential distribution owing to their common history [17], which can mislead species delimitation tools that rely on a single non-recombining locus and so result in taxonomic over-splitting [18,19]. By contrast, non-neutral alleles are lost or go to fixation more rapidly at a rate proportional to their selection coefficient and so are less likely to reveal high diversity. High genetic diversity within a single population sample could potentially be explained by balancing selection if fitness was determined by allele frequency in the population. In the case of mtDNA sequences, balancing selection is possible if the variation involves expressed differences. Comparing the number of synonymous changes with the number of non-synonymous changes allows inference of the likely selection process [20]. Neutral genetic variation can be explained by population size in the absence of evidence of other processes.

The third and fourth processes leading to a putative single population sample containing relatively high genetic diversity are cryptic species [21] and recent introgression [22]. Elegant population genetics tools are available to differentiate between these possible sources of genetic diversity if information is available from more than one marker [23–28].

Morphological variation derived from genotypic variation can provide the information needed to determine whether high genetic diversity results from sampling more than one species [21]. Concordance of characters and the absence of intermediates provide evidence for multiple genotypic clusters (species; [29]). Independent populations would show linkage of alleles at independent loci and a lack of heterozygote individuals. Infection with the parasitic bacteria *Wolbachia* could result in reproductive incompatibility between individuals infected and uninfected or between individuals infected with different bacteria stains [30]. In the long term, this would be expected to result in concordance of mtDNA haplotypes and nuclear alleles and a deficiency of heterozygote individuals; however, recent invasion of *Wolbachia* strains might resemble introgression. Maternally transmitted parasites are genetically linked to the mitochondria and if they cause cytoplasmic incompatibility will result in a loss of mtDNA diversity [31]. The presence of cryptic species provides no expectation of geographical limits, but hybridization is usually geographically constrained [32,33], producing a restricted region or zone of high diversity. Within such regions, bimodal distributions of genetic and/or phenotypic characters are indicative of selection against hybrids [25]. In the case where high mtDNA diversity in a putative population arises from hybridization, an association of unlinked markers would be expected.

In New Zealand, many endemic invertebrates have been subjects of phylogeographical investigations, with their mtDNA genetic diversity used to infer historical range changes [34,35]. Interpretation of notably high levels of mtDNA sequence variation within samples varies (reviewed in Trewick *et al*. [35]). For example, mtDNA differences have been ascribed to an accelerated rate of mtDNA evolution [36], or ancient (Pliocene) subdivision of populations and subsequent hybridization [37]. The contrast between Southern and Northern hemisphere study systems is striking. Low diversity in many invertebrate species in Europe and North America is concordant with the recent absence of suitable habitat owing to southward extension of polar ice sheets during the last glacial maximum (approx. 32 000–28 000 years ago; [38–41]). Despite large modern species' ranges, low diversity in these areas is inferred as resulting from recent range expansion [23]. By contrast, although smaller, New Zealand lost disproportionally less terrestrial habitat to snow and ice, and increased land area from lower sea level plus relatively benign climate owing to maritime amelioration ensured widespread survival of forest/scrub habitat [42–44].

**Figure 1.**
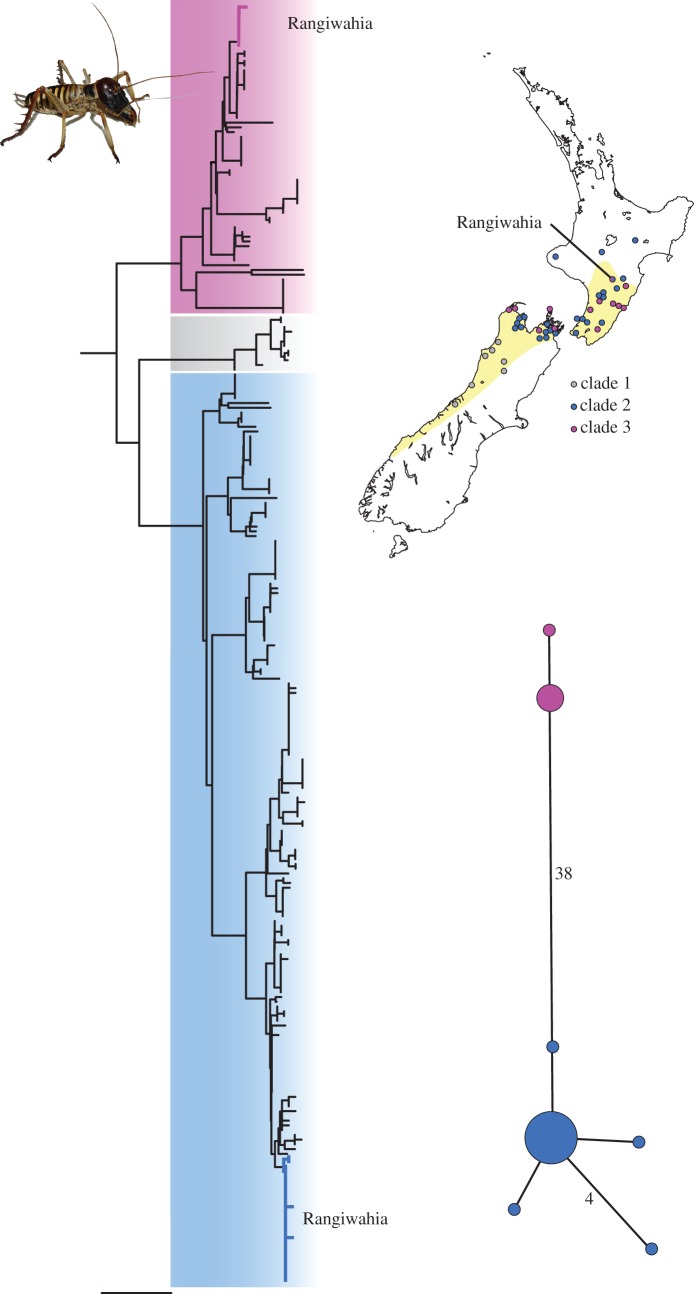
Spatial distribution of mtDNA haplotype diversity within the New Zealand tree wētēt *H. crassidens* (yellow). A network of all mtDNA haplotypes observed within a single population sample (Rangiwahia) illustrates difference between DNA sequences and their relative frequency (circle area is scaled to sample size).

One endemic insect, the tree wētā *Hemideina crassidens* (Orthoptera; Ensifera; Anostostomatidae), was probably widespread in North Island New Zealand during the last glacial maximum [44]. This arboreal wētā species contains three distinct lineages of mtDNA haplotypes (figure 1) with average genetic distances among haplogroups of 0.07–0.09 (GTR model with variable base frequencies and symmetrical substitution matrix) and much higher levels than typically accepted within species [1]. One *H. crassidens* mtDNA haplogroup is restricted to wētā samples from the southern part of the species’ range, concordant with a distinct 19-chromosome race [45]. Two others (haplogroups 2 and 3) have overlapping occurrence in South and North Island *H. crassidens* (figure 1). Both mtDNA haplogroups 2 and 3 occur within the 15-chromosome race of this insect [44]. In a separate study, we sought evidence of *Wolbachia* infections in a range of New Zealand insect species. We did not detect *Wolbachia*-like DNA sequences in any sampled population of *Hemideina* species, including individuals from both mtDNA haplogroups 2 and 3 of *H. crassidens* [46].

Our aim was to determine what biological process most likely led to the high mtDNA diversity within the 15-chromosome race of *H. crassidens*. We looked for spatial structure in the mtDNA diversity over the full landscape of the species for evidence of partitions in the distribution of genetic variation that would be a signature of past isolation followed by hybridization. We then focused on a sample from a single location where both lineages are present. By examining nuclear markers, we sought evidence of partitioning of nuclear genetic variation concordant with mtDNA variation that would support a hypothesis of two species. We compared the rate of silent substitutions to expressed substitutions using the whole mtDNA genome to detect any signature of selection.

## 2. Material and methods

### 2.1. Spatial structure of mtDNA diversity within *Hemideina crassidens*

The distribution of mitochondrial sequence variation within the natural range of *H. crassidens* was explored to identify potential spatial structure that might arise from historical processes or selection (data published in Bulgarella *et al*. [44]). We examined the distribution of the three distinct mitochondrial haplogroups in relation to both latitude and elevation. We visualized genetic distance patterns across the complete range of *H. crassidens* using three-dimensional surface plots known as ‘genetic landscape shapes' using Alleles In Space (AIS, http://www.marksgeneticsoftware.net/, [47]). To do this, a connectivity network of sampled individuals was constructed and genetic distances (*Z_i_*, calculated as the average proportion of nucleotide differences between individuals from different sampling areas) assigned to landscape coordinates at midpoints (*X_i_*, *Y_i_*) of the connectivity edges, as described in Miller *et al*. [48]. Genetic distances were then inferred using an interpolation procedure on a uniformly spaced grid overlaid on the sampled landscape. Finally, a three-dimensional surface plot where *X* and *Y* coordinates correspond to geographical locations (latitude and longitude) on a rectangular grid and surface plot heights (*Z*) reflecting genetic distances was generated [49,50], We selected a 50 × 50 grid and a distance weighting parameter (*a*) of 1. Additional analyses were performed using a variety of grid sizes (50 × 50, 60 × 60, 100 × 100), distance weighting parameters (*a* = 0.5–2) and raw and residual genetic distances to make sure that interpretations were not sensitive to these parameters, as recommended.

High nucleotide diversity (owing to the coexistence of haplotypes from two distinct mitochondrial haplogroups) was recorded in a tree wētā sample from Rangiwahia Forest Park (39°53′38.51^″^ S; 176°0′10.80^″^ E) in central North Island New Zealand [44]. Additional specimens were collected at this location for the present analysis. A random sample of wētā with respect to age and sex was extracted from their daytime refuge holes in tree branches. Authority to collect came from the New Zealand Department of Conservation (TW-32116-FAU). All individuals were identified as *H. crassidens* using a combination of colour and spine traits that differentiates this species from other *Hemideina* species [51].

### 2.2. Morphological variation

Two morphological traits were recorded for each individual: total number of stridulatory ridges and total number of hind tibia dorsal spines. Neither of these traits correlates with wētā instar, and therefore, wētā at any age can be included in the analysis. Tree wētā use their abdominal stridulatory ridges to make rasping sounds when rubbed against pegs on the retrolateral surface of the hind femur, probably for intraspecific communication [52]. Ridge numbers vary within and among tree wētā species [53]. The number of ridges on the left and right sides of the abdomen was combined. The number of prolateral hind tibia spines differentiates *H. crassidens* and *Hemideina thoracica* at some locations suggesting interspecific selection. We therefore recorded all dorsal hind tibia spines (left and right) for each individual at Rangiwahia. Tubercles with rounded ends rather than points (as seen in spines) were recorded as half spines (0.5) if they occurred in the normal position for a dorsal tibia spine.

### 2.3. mtDNA analysis

Genomic DNA was extracted from each individual and an approximately 800 bp mtDNA fragment amplified, as previously described [44]. The mtDNA fragment spanning part of cytochrome *b* (*cytb*), tRNA serine and part of NADH dehydrogenase 1 (*ND1*) sequences were checked, edited and aligned in Geneious v. 8.1.4 (http://www.geneious.com, [54]). We calculated average nucleotide site diversity (*π*) for the population sample using the method of Tajima [55], with an alignment of mtDNA sequence and tools provided in Geneious. We estimated population size using the equation *N* = *π*/4*u* [13]. Three mutation rate estimates from published work were used to calculate population size: *Drosophila* rate of 6.2 × 10^−8^ [56]; *Daphnia* 1.5 × 10^−7^ [57] and *Nematode* 7.6 × 10^−8^ [58]. The tRNASer sequence within the mtDNA fragment was removed from the dataset before networks were inferred. Deletions and insertions (INDELS) in this tRNA region differentiate the haplotypes, but this variation is not readily incorporated into network analysis nor estimates of genetic distance. A minimum spanning network [59] was inferred with PopArt [60] using 29 sequences, trimmed to 519 bp. Aligned mtDNA sequences are available at http://evolves.massey.ac.nz/Data/Rangiwahia%20weta.zip and http://dx.doi.org/10.5061/dryad.rg15p [61].

### 2.4. Nuclear markers

One coding, putatively neutral nuclear locus (Sperm flagallar protein; Sflag; [62]) was amplified and sequenced, and coded as genotype data for analysis in combination with microsatellite loci. The 400 bp fragment of the nuclear gene (Sflag) was amplified and sequenced using the primers TWnucSflagF (5′-TCGCCAGTTCAGACCTAGGATGAGG-3′) and TWnucSflagR (5′-TGGCTCTGTACAAGGCTGGGA-3′) [62]. Only two Sflag alleles were detected and as some wētā were homozygous at this locus, heterozygote individuals could be identified and genotyped as they contained two nucleotides at each of the three sites that differentiate the two alleles.

Four microsatellite loci (HM04, HR12, HR35 and HR43) were amplified using primers and PCR conditions developed for other species within the genus *Hemideina* [63,64]. One primer from each pair was fluorescently labelled with either 6-FAM, PET or VIC dyes (Applied Biosystems). Fluorescent DNA fragments including a size standard were detected using an ABI 3130xl genetic analyser (Applied Biosystems) and sized with the Geneious microsat plugin and individuals manually genotyped (data available at http://evolves.massey.ac.nz/Data/Rangiwahia%20weta.zip and http://dx.doi.org/10.5061/dryad.rg15p) [61].

Evidence of linkage disequilibrium and deviations from the Hardy–Weinberg equilibrium were examined using Genepop v. 4.2 [65,66], with the following Markov chain parameters: 10 000 dememorization, 100 batches and 10 000 iterations per batch. Evidence of linkage disequilibrium used probability tests with the Bonferroni correction for multiple tests (*n* = 10; *p* < 0.005). Where two species are sympatric, but rarely or never interbreeding, alleles at independent loci are expected to show linkage disequilibrium as heterozygote individuals will be scarce or absent. With our wētā sample treated as a single population sample, detection of heterozygote deficiency would indicate non-random mating.

With the data partitioned between wētā from mtDNA haplogroup 2 (*n* = 23) and wētā haplogroup 3 (*n* = 6), we looked for evidence for these being non-interbreeding sympatric populations (e.g. different species). If distinct, differentiation at nuclear loci would result in significant departure of *F*_ST_ from zero. Pairwise *F*_ST_ was estimated using genotypes at five nuclear loci with Genepop v. 4.2.

### 2.5. Whole mtDNA selection

Two individuals, representing two mtDNA lineages (haplogroups 2 and 3), were used to calculate the *K*_A_/*K*_S_ ratio in the coding regions of the mtDNA using both maximum-likelihood and approximate methods with KaKs_Calculator [67]. The ratio of non-synonymous substitutions (*K*_A_) to synonymous substitutions (*K*_S_) identifies the selective pressures occurring in protein-coding genes; a ratio of 1 implies neutral selection, greater than 1 implies positive selection and less than 1 implies constraining (purifying) selection. Constraining selection is predicted for coding DNA in large populations. Total genomic DNA from two individuals (*H. crassidens* haplogroup 2 (North) and *H. crassidens* haplogroup 3 (South)) were separately processed through massive parallel, high-throughput sequencing (Illumina HiSeq 2000) to obtain whole mitochondrial sequences for a separate phylogenetic study [68]. Genomic DNA was fragmented, prepared using the ThruPLEX® DNA-seq Kit (Rubicon Genomics) and used to generate 100 bp paired-end sequence. Adapter sequence barcodes and poor-quality data were removed from Illumina sequence reads using Cutadapt [69]. The quality of the data was assessed using SolexaQA [70] before *de novo* assembly with Velvet v. 1.1 [71] and Abyss v. 2.3.3 [72] using a *k*-mer length of 49. Contig files were compared to annotated Orthoptera mitochondrial sequences available on GenBank (NCBI). We found evidence of nuclear copies of some regions of the mtDNA and were able to remove paralogs using relative copy number and protein translation. The resulting assembled mitochondrial genomes were 16 535 and 15 905 bp in length (total 11 199 bp of coding regions) with the same gene order previously identified in Ensifera [73], but with the inclusion of a repetitive unit of several hundred bp in length between tRNAQ and *cox1*. Fasta files of the two *H. crassidens* mitochondrial genome sequences can be downloaded from http://evolves.massey.ac.nz/Data/Rangiwahia%20weta.zip and http://dx.doi.org/10.5061/dryad.rg15p [61].

### 2.6. Population genetic analysis with principal components analysis

Principal components analysis (PCA) was performed on the morphological and genetic data combined (excluding mtDNA). Stridulatory ridge counts and tibia spine counts were each treated as a single trait (range 9–15 ridges; 18.5–22 spines). For nuclear loci, we treated each allele as a character trait and each individual was given a character state per allele: 0 (if absent), 1 or 2 (if homozygous), as recommended by Odong *et al*. [74]. Alleles within a locus are not independent; thus, a PCA is an appropriate dimensionality reduction tool. PCA was performed with standardization using the function *prcomp* in R package ‘STATS’ (R Development Core Team, 2008) (filename, centre = FALSE, scale = TRUE). The first five principal components (cumulative proportion of variance was 0.664) were used in a model-based clustering approach using the Bayesian information criterion to select the number of genotypic clusters in the R package ‘Mclust’ [75,76]. Model-based clustering was used to classify individual wētā without prior information about the number of clusters or specimen identity [75]. The Mclust algorithm is built from a general model, where the total dataset is considered as a mixture of multivariate normal datasets, with a selection of covariance structures and vectors of expectation [77]. The Mclust analysis examined 90 models (10 different models with various combinations of parametrization and one to nine clusters/components). The optimal model was selected based on the maximized log likelihood, with a penalty for the number of parameters in the model [75,77]. We compared the concordance of clusters resolved from mitochondrial haplogroups with nuclear data (phenotype and microsatellite genotypes) using Cohen's *κ*. Cohen's *κ* coefficient measures agreement of assignment to categories, where values between 0.75 and 1 indicate non-random agreement, but where there is no agreement among the methods other than what would be expected by chance *κ* ≤ 0 (online statistical calculator: https://www.niwa.co.nz/node/104318/kappa).

## 3. Results

**Figure 2.**
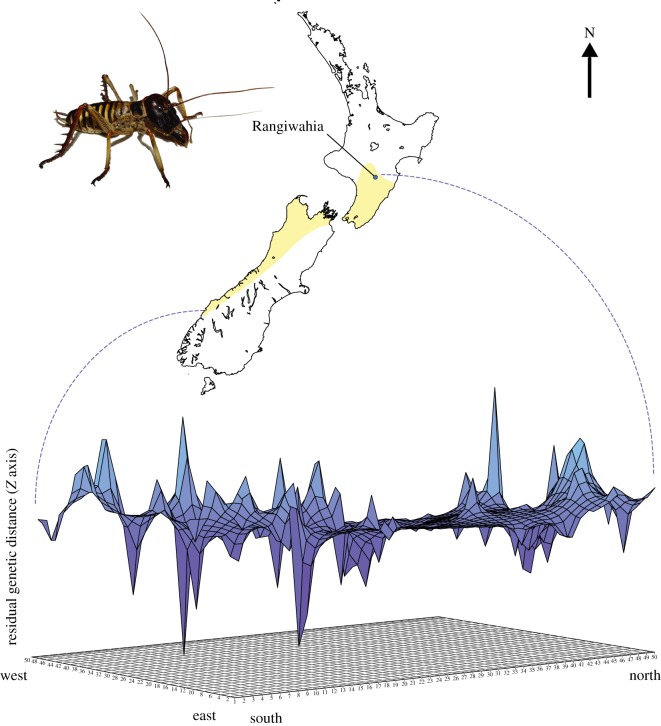
Spatial distribution of mtDNA diversity within the New Zealand tree wētā *H. crassidens* across its natural range (yellow shading). Genetic landscape *X* and *Y* axes correspond to geographical locations of population samples within the overall physical landscape examined for the whole range of *H. crassidens*. Surface plot heights reflect relative genetic distances, data from Bulgarella *et al*. [44].

High nucleotide diversity owing to the coexistence of three distinct mitochondrial haplotype lineages has been described within the natural range of *H. crassidens* (figure 1*a*; Appendix S1 of [35]; jbi12224-sup-0001-Appendix S1–S3.docx). Examination of spatial structure in this mtDNA diversity revealed some evidence of geographical structure. Haplogroup 1 is concordant with chromosome race 19 and found only south of −41^o^5′, whereas no evidence of latitudinal or elevation partitioning was observed among haplotypes in haplogroups 2 and 3. This absence of spatial structure through the range of *H. crassidens* was well illustrated by genetic landscape shape interpolation (figure 2).

‘Peaks’ indicating greatest genetic distances among population samples were scattered through the species range. Qualitatively similar results were obtained using various grid sizes and a range of distance weighting parameters (*a* = 0.5–2). Thus, we obtained no evidence of the spatial structure that would be expected from secondary contact between previously isolated populations, nor evidence of barriers or discontinuities within the range of mtDNA haplogroups 2 and 3.

We increased sampling of *H. crassidens* at a location where haplotypes from both haplogroups 2 and 3 had been recorded (Rangiwahia, figure 2). Our 702 bp mtDNA fragment resolved 10 haplotypes from 29 individuals (data available at: http://evolves.massey.ac.nz/Data/Rangiwahia%20weta.zip and http://dx.doi.org/10.5061/dryad.rg15p; [61]). Average nucleotide site diversity (*π*) for this population sample was high (0.1035). Using this nucleotide site diversity, estimates of population size ranged from 172 500 to 417 338 depending on mutation rate used. The only available insect example (*Drosophila* mtDNA) has the lowest mutation rate and thus gave the largest population size estimate.

**Figure 3.**
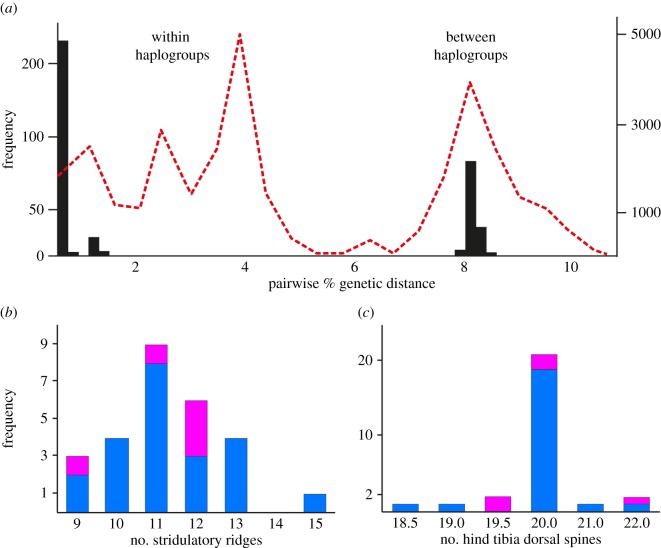
The non-recombining mtDNA has a bimodal frequency distribution of pairwise differences generated by the coalescent process in a large population, for whole *H. crassidens* species dataset (dotted red line), and Rangiwahia population sample (black fill) (*a*). Unimodal frequency distributions of morphological traits suggest that hybridization is not the cause of high mtDNA variation within a single population sample of the New Zealand wētā *H. crassidens*. Variation in numbers of stridulatory ridges (*b*) and spines on hind tibia (*c*) in Rangiwahia population sample are not concordant with mtDNA lineages of specimens; haplogroup 2 (blue) and haplogroup 3 (pink).

For further analyses, we trimmed the sequences to include only protein-coding sections, which removed tRNA INDELS. Resulting mtDNA sequences resolved seven haplotypes attributable to haplogroup 2 or 3 of the previously reported *H. crassidens* diversity [44] (figure 1). These haplotypes differed by a maximum of 7.9% (uncorrected; 41 substitutions out of 519 bp; figure 1), with an average uncorrected difference of 2.7% (14.2 substitutions). The frequency distribution of pairwise genetic differences within the Rangiwahia haplotype sample was bimodal as observed for the whole species dataset (figure 3*a*). When translated, nucleotide substitutions resulted in three amino acid replacements that could be subject to natural selection. However, when we calculated the *K*_a_/*K*_s_ ratio for all coding regions of the whole mitochondrial genome (representing the two mtDNA clades; (http://evolves.massey.ac.nz/Data/Rangiwahia%20weta.zip and http://dx.doi.org/10.5061/dryad.rg15p [61])), the result was less than 1 (0.03), implying constraining selection not positive balancing selection was occurring in the mtDNA.

Each wētā from the Rangiwahia population sample was genotyped at five polymorphic nuclear loci. The number of alleles per locus ranged from 2 to 12 (table 1). None of the five nuclear loci showed evidence of linkage to any other locus (*p*-values ranged between 1 and 0.021). Hardy–Weinberg expectations were met with no evidence of homozygote or heterozygote excess (multi-locus exact tests: heterozygote deficit *p* = 0.9321; heterozygote excess *p* = 0.0679). The two wētā haplogroups showed no evidence of genetic differentiation at nuclear loci. Population pairwise *F*_ST_ = −0.01777; genotypic differentiation was 0 (*p* = 0.895965 exact *G* test; *p* = 0.897884 Fisher's method).

**Table 1.** Genetic diversity within a single population sample of the tree wētā *H. crassidens* for five nuclear markers. (Sample is divided using the two distinct mtDNA haplogroups observed. The number of alleles per locus is indicated along with (number) of alleles restricted to each mtDNA haplogroup. *n* = sample size.)

	nuclear loci					
MtDNA	*n*	*Sflag*	*HR12*	*HR35*	*HR43*	*HM04*
haplogroup 2	23	2	2	12 (6)	3 (1)	3 (1)
haplogroup 3	6	2	2	6	2	3 (1)

No differences between phenotypic traits were observed when compared for the two haplogroups. Stridulatory ridge counts averaged 11.2 and 11.3, and spine counts on hind tibia averaged 20.02 and 20.20 (*t*-test *p* > 0.05). Frequency distributions of these traits were unimodal (figure 3). Combining phenotypic traits and nuclear markers in PCA, principal components 1 and 2 (45.5% of variation) showed no concordance with mtDNA haplogroup (table 2). Although two genotypic clusters were naively resolved using Bayesian modelling in Mclust with the first five principal components (66.4% of variation), these clusters were not concordant with mtDNA haplogroups (Cohen's *κ* estimate was −0.050). Furthermore, the clusters inferred for just PC1 and PC2 did not coincide with the clusters resolved using PC1–5 (Cohen's *κ* −0.088).

**Table 2.** Assignment of wētā individuals to clusters based on mtDNA haplogroup is not concordant with cluster assignment based on Bayesian modelling using principal components of phenotypic and genotypic variation. (Alternative assignments indicated by italics.)

			mtDNA	assigned cluster	
wētā ID	sex	age	haplogroup	PC 1 and 2	PC 1–5
Hc-Ran-618	female	juvenile	*2*	*2*	1
Hc-Ran-624	female	adult	*2*	*2*	1
Hc-Ran-619	male	adult	*2*	1	1
Hc-Ran-620	female	adult	*2*	*2*	1
Hc-Ran-1308	female	adult	*2*	1	*2*
Hc-Ran-1310	male	juvenile	*2*	1	1
Hc-Ran-1311	female	juvenile	*2*	1	1
Hc-Ran-1312	female	juvenile	*2*	1	1
Hc-Ran-1328	female	adult	*2*	1	1
Hc-Ran-1315	female	adult	*2*	*2*	1
Hc-Ran-622	female	adult	*2*	*2*	1
Hc-Ran-1316	male	juvenile	*2*	3	*2*
Hc-Ran-1317	male	juvenile	*2*	1	1
Hc-Ran-1318	male	juvenile	*2*	1	1
Hc-Ran-1319	male	juvenile	*2*	1	1
Hc-Ran-1320	female	juvenile	*2*	1	1
Hc-Ran-1321	female	juvenile	*2*	1	1
Hc-Ran-1322	female	juvenile	*2*	1	*2*
Hc-Ran-1323	male	juvenile	*2*	1	*2*
Hc-Ran-1324	female	juvenile	*2*	1	1
Hc-Ran-1325	female	juvenile	*2*	1	*2*
Hc-Ran-1326	male	juvenile	*2*	1	1
Hc-Ran-1327	male	juvenile	*2*	1	1
Hc-Ran-NN	female	juvenile	3	1	*2*
Hc-Ran-621	female	adult	3	1	1
Hc-Ran-1309	male	adult	3	1	1
Hc-Ran-1313	female	juvenile	3	1	1
Hc-Ran-1314	female	adult	3	1	1
Hc-Ran-623	male	adult	3	*2*	1

## 4. Discussion

We confirmed that the population sample of tree wētā from Rangiwahia contains high mtDNA diversity, resulting from the presence of individuals at the same location with very different haplotypes. We focused on this site because sympatry of the mtDNA haplogroups allowed for detection of separate species (should they exist) using genotypic information [29]. We considered four biological processes that might result in the high mtDNA diversity within our sampling of *H. crassidens* tree wētā. Secondary contact and hybridization was not supported by the spatial distribution of mtDNA lineages 2 and 3 throughout the species range. A lack of genetic linkage, presence of heterozygotes and lack of genetic differentiation (*F*_ST_ = 0) are contrary to a hypothesis of cryptic species. Mito-nuclear discordance owing to intercellular bacteria can be excluded as an explanation as parasitic *Wolbachia* is not found in this genus [46]. Using a combination of morphological characters and nuclear markers, we observed no concordance among clusters inferred from different datasets (mtDNA, nuclear). Although one of the two mtDNA haplogroups at Rangiwahia was represented by just six specimens, this does not appear to have prevented us from detecting population structure. For example, our estimate of genotypic differentiation (*F*_ST_) based on five loci was negative, which does not suggest a type II error, and our tests for significant deviations incorporated sample size. With the same sample size, the best model to explain the phenotypic and genotypic variation (using the Bayesian cluster-based approach) resolved more than one group, suggesting that our sample was sufficient to detect variation, had it existed.

Although some mtDNA sequence differences were expressed, we detected constraining selection within the mitochondrial genome as a whole, rather than diversifying selection that would be more likely if balancing selection was maintaining the high mtDNA diversity [78,79]. We studied the whole mtDNA genome so can exclude the possibility of diversifying selection in other regions of this (non-recombining) molecule. Therefore, retention of a large population is the most likely explanation for the genetic diversity observed here within *H. crassidens*. In a large population, a non-recombining gene without selection will tend to yield a distribution of pairwise differences that deviates substantially from the geometric distribution expected in a population with constant size. Pairwise differences of mtDNA sequence frequently show a bimodal or trimodal distribution simply because the history of coalescent events imposes a substantial correlation [17]. Simulation studies of mtDNA species delimitation tools find that over-splitting is a problem with medium-sized and large populations [18] owing to this natural correlation using a non-recombining gene sequence. When more complex population processes such as recent population decline are incorporated into simulations, the excess of recent coalescent events also creates mtDNA clusters within species. These clusters have pairwise differences with bimodal frequency distributions, such ‘barcoding gaps’ could be misidentified as evidence of separate taxa as observed by Fujisawa & Barraclough [19]. Thus, the apparent ‘barcoding gap’ observed in *H. crassidens* mtDNA could be the result of a large population accentuated by recent decline resulting from destruction of forest habitat, or range expansion with leading edge effects.

Our estimates of population size for this tree wētā of 170–420 thousand are plausible for a herbivorous ectotherm. *Hemideina crassidens* was probably abundant throughout the Pleistocene (2.58 Myr). Evidence from niche modelling and patterns of genetic diversity suggest that *H. crassidens* retained large population sizes in New Zealand during the climate cycling of the last 40 K years [44]. These most recent cycles overwrote the signature for earlier cycles, but climate modelling suggests that the species would not have been significantly reduced in range size during any of the Milankovitch cycles. Competitive interactions are likely to have had an influence on realized niche, but these probably did not limit population size substantially [39].

Sequence divergence observed between the three distinct mtDNA lineages within *H. crassidens* could be used to infer that the mtDNA lineages shared a recent common ancestor approximately 3–4 Ma into the Pliocene. Although the shape of New Zealand during the Pliocene was considerably different from that of the current islands [42], this has little relevance for the current distribution of mtDNA haplotypes observed today. However, we cannot exclude the possibility that past New Zealand islands drove divergence by vicariance, followed by secondary contact and mixing so that only the distinct mtDNA haplotypes are retained from the parental populations. It is tempting to speculate that during range expansion (either northwards during cool phases or south during warming), smaller effective population size might have resulted in coalescent events that generated the appearance of a ‘barcoding gap’. MtDNA haplogroup 1 is restricted to recently colonized southern sites—suggesting that leading edge effects yielded shallow coalescence of this mtDNA cluster within the species. As both haplogroups 2 and 3 are widespread, the contrast between old and recent coalescence is not associated with the most recent range shifts. However, it is not necessary to evoke vicariance or changes in population size as simulation studies demonstrate that a common history for a non-recombining gene results in distributions exactly as observed here [17].

We have shown how hypotheses that arise from large mtDNA sequence differences can be tested using phenotypic and nuclear genetic data. As there is no evidence of balancing selection or that these mtDNA differences represent biological lineages with separate histories, we suggest that large population size explains the retention of high diversity. The origin of the divergent mtDNA haplogroups might result from complex biogeographical scenarios or they might simply represent normal, stochastic processes of mutation and extinction of a non-recombining locus within a large population as demonstrated by simulation studies [17,18]. We urge others to consider population size rather than cryptic species as a null hypothesis when a lack of nuclear or phenotypic data prevent testing of alternative explanations.

## Data accessibility

Data can be downloaded from the following sites: jbi12224-sup-0001-Appendix S1–S3.docx. http://evolves.massey.ac.nz/Data/Rangiwahia%20weta.zip. http://dx.doi.org/10.5061/dryad.rg15p [61].

## References

[1] Hebert PDN, Cywinska A, Ball SL, deWaard JR 2003 Biological identifications through DNA barcodes. Proc. R. Soc. Lond. B 270, 313–321. (doi:10.1098/rspb.2002.2218) 10.1098/rspb.2002.2218 PMC1691236 12614582

[2] Krishna Krishnamurthy P, Francis RA 2012 A critical review on the utility of DNA barcoding in biodiversity conservation. Biodivers. Conserv. 21, 1901–1919. (doi:10.1007/s10531-012-0306-2)

[3] Collins RA, Cruickshank RH 2012 The seven deadly sins of DNA barcoding. Mol. Ecol. Resour. 13, 969–975. 23280099 10.1111/1755-0998.12046

[4] Bell KL, Burgess KS, Okamoto KC, Aranda R, Brosi BJ 2016 Review and future prospects for DNA barcoding methods in forensic palynology. Forensic Sci. Int. Genet. 21, 110–116. (doi:10.1016/j.fsigen.2015.12.010) 26751251 10.1016/j.fsigen.2015.12.010

[5] Hopken MW, Orning EK, Young JK, Piaggio AJ 2016 Molecular forensics in avian conservation: a DNA-based approach for identifying mammalian predators of ground-nesting birds and eggs. BMC Res. Notes. 9, 14 (doi:10.1186/s13104-015-1797-1) 26738484 10.1186/s13104-015-1797-1 PMC4704294

[6] Sharma R, Stuckas H, Bhaskar R, Rajput S, Khan I, Goyal SP, Tiedemann R 2009 MtDNA indicates profound population structure in Indian tiger (*Panthera tigris tigris*). Conserv. Genet. 10, 909–914. (doi:10.1007/s10592-008-9568-3)

[7] Darling JA, Blum MJ 2007 DNA-based methods for monitoring invasive species: a review and prospectus. Biol. Invasions. 9, 751–765. (doi:10.1007/s10530-006-9079-4)

[8] Valentini A et al. 2009 New perspectives in diet analysis based on DNA barcoding and parallel pyrosequencing: the *trn* L approach. Mol. Ecol. Resour. 9, 51–60. (doi:10.1111/j.1755-0998.2008.02352.x) 10.1111/j.1755-0998.2008.02352.x 21564566

[9] Moritz C, Cicero C 2004 DNA barcoding: promise and pitfalls. PLoS Biol. 2, e354 (doi:10.1371/journal.pbio.0020354) 15486587 10.1371/journal.pbio.0020354 PMC519004

[10] Rubinoff D, Cameron S, Will K 2006 A genomic perspective on the shortcomings of mitochondrial DNA for ‘barcoding’ identification. J. Hered. 97, 581–594. (doi:10.1093/jhered/esl036) 17135463 10.1093/jhered/esl036

[11] Trewick SA 2008 DNA barcoding is not enough: mismatch of taxonomy and genealogy in New Zealand grasshoppers (*Orthoptera*: *Acrididae*). Cladistics 24, 240–254. (doi:10.1111/j.1096-0031.2007.00174.x)

[12] Kimura M 1968 Evolutionary rate at the molecular level. Nature 217, 624–626. (doi:10.1038/217624a0) 5637732 10.1038/217624a0

[13] Charlesworth B 2009 Fundamental concepts in genetics: effective population size and patterns of molecular evolution and variation. Nat. Rev. Genet. 10, 195–205. (doi:10.1038/nrg2526) 19204717 10.1038/nrg2526

[14] Ashfaq M, Prosser S, Nasir S, Masood M, Ratnasingham S, Hebert PDN 2015 High diversity and rapid diversification in the head louse, *Pediculus humanus* (*Pediculidae: Phthiraptera*). Sci. Rep. 5, 14188 (doi:10.1038/srep14188) 26373806 10.1038/srep14188 PMC4570997

[15] Page RD, Lee PL, Becher SA, Griffiths R, Clayton DH 1998 A different tempo of mitochondrial DNA evolution in birds and their parasitic lice. Mol. Phylogenet. Evol. 9, 276–293. (doi:10.1006/mpev.1997.0458) 9562986 10.1006/mpev.1997.0458

[16] Toon A, Hughes JM 2008 Are lice good proxies for host history? A comparative analysis of the Australian magpie, *Gymnorhina tibicen*, and two species of feather louse. Heredity 101, 127–135. (doi:10.1038/hdy.2008.37) 18461081 10.1038/hdy.2008.37

[17] Slatkin M, Hudson R 1991 Pairwise comparisons of mitochondrial DNA sequences in stable and exponentially growing populations. Genetics 129, 555–562. 1743491 10.1093/genetics/129.2.555 PMC1204643

[18] Dellicour S, Flot J-F 2015 Delimiting species-poor data sets using single molecular markers: a study of barcode gaps, haplowebs and GMYC. Syst. Biol. 64, 900–908. (doi:10.1093/sysbio/syu130) 25601944 10.1093/sysbio/syu130

[19] Fujisawa T, Barraclough TG 2013 Delimiting species using single-locus data and the generalized mixed yule coalescent approach: a revised method and evaluation on simulated data sets. Syst. Biol. 62, 707–724. (doi:10.1093/sysbio/syt033) 23681854 10.1093/sysbio/syt033 PMC3739884

[20] Nekrutenko A, Makova KD, Li W 2002 The KA/KS ratio test for assessing the protein-coding potential of genomic regions: an empirical and simulation study. Genome Res. 12, 198–202. (doi:10.1101/gr.200901) 11779845 10.1101/gr.200901 PMC155263

[21] Bickford D, Lohman DJ, Sodhi NS, Ng PKL, Meier R, Winker K, Ingram KK, Das I 2007 Cryptic species as a window on diversity and conservation. Trends Ecol. Evol. 22, 148–155. (doi:10.1016/j.tree.2006.11.004) 17129636 10.1016/j.tree.2006.11.004

[22] Mallet J 2008 Hybridization, ecological races and the nature of species: empirical evidence for the ease of speciation. Phil. Trans. R. Soc. B 363, 2971–2986. (doi:10.1098/rstb.2008.0081) 18579473 10.1098/rstb.2008.0081 PMC2607318

[23] Excoffier L, Foll M, Petit RJ 2009 Genetic consequences of range expansions. Annu. Rev. Ecol. Evol. Syst. 40, 481–501. (doi:10.1146/annurev.ecolsys.39.110707.173414)

[24] Baird SJE 2015 Exploring linkage disequilibrium. Mol. Ecol. Resour. 15, 1017–1019. (doi:10.1111/1755-0998.12424) 26261040 10.1111/1755-0998.12424

[25] Jiggins CD, Mallet J 2000 Bimodal hybrid zones and speciation. Trends Ecol. Evol. 15, 250–255. (doi:10.1016/S0169-5347(00)01873-5) 10802556 10.1016/s0169-5347(00)01873-5

[26] Barton NH, Gale KS 1993 Genetic analysis of hybrid zones. In Hybrid zones and the evolutionary process (ed. Harrison RG), pp. 13–45. New York, NY: Oxford University Press.

[27] Bulgarella M, Trewick SA, Godfrey AJR, Sinclair BJ, Morgan-Richards M 2015 Elevational variation in adult body size and growth rate but not in metabolic rate in the tree weta *Hemideina crassidens*. J. Insect. Physiol. 75, 30–38. (doi:10.1016/j.jinsphys.2015.02.012) 25753546 10.1016/j.jinsphys.2015.02.012

[28] Toews DPL, Brelsford A 2012 The biogeography of mitochondrial and nuclear discordance in animals. Mol. Ecol. 21, 3907–3930. (doi:10.1111/j.1365-294X.2012.05664.x) 22738314 10.1111/j.1365-294X.2012.05664.x

[29] Mallet J 1995 A species definition for the modern synthesis: trends in ecology and evolution. Trends Ecol. Evol. 10, 294–299. (doi:10.1016/0169-5347(95)90031-4) 21237047 10.1016/0169-5347(95)90031-4

[30] Werren JH, Baldo L, Clark ME 2008 *Wolbachia*: master manipulators of invertebrate biology. Nat. Rev. Microbiol. 6, 741–751. (doi:10.1038/nrmicro1969) 18794912 10.1038/nrmicro1969

[31] Jiggins FM 2003 Male-killing *Wolbachia* and mitochondrial DNA: selective sweeps, hybrid introgression and parasite population dynamics. Genetics 164, 5–12. 12750316 10.1093/genetics/164.1.5 PMC1462540

[32] Barton NH, Hewitt GM 1985 Analysis of hybrid zones. Annu. Rev. Ecol. Syst. 16, 113–148. (doi:10.1146/annurev.es.16.110185.000553)

[33] Endler J 1977 Geographical variation, speciation and clines. Monogr. Popul. Biol. 10, 1–246. 409931

[34] Wallis GP, Trewick SA 2009 New Zealand phylogeography: evolution on a small continent. Mol. Ecol. 18, 3548–3580. (doi:10.1111/j.1365-294X.2009.04294.x) 19674312 10.1111/j.1365-294X.2009.04294.x

[35] Trewick SA, Wallis GP, Morgan-Richards M 2011 The invertebrate life of New Zealand: a phylogeographic approach. Insects 2, 297–325. (doi:10.3390/insects2030297) 26467729 10.3390/insects2030297 PMC4553545

[36] Boyer SL, Baker JM, Giribet G 2007 Deep genetic divergences in *Aoraki denticulata* (Arachnida, Opiliones, Cyphophthalmi): a widespread ‘mite harvestman’ defies DNA taxonomy. Mol. Ecol. 16, 4999–5016. (doi:10.1111/j.1365-294X.2007.03555.x) 17944852 10.1111/j.1365-294X.2007.03555.x

[37] Morgan-Richards M, Trewick SA, Wallis GP 2001 Chromosome races with Pliocene origins: evidence from mtDNA. Heredity 86, 303–312. (doi:10.1046/j.1365-2540.2001.00828.x) 11488967 10.1046/j.1365-2540.2001.00828.x

[38] Williams PW, McGlone M, Neil H, Zhao J-X 2015 A review of New Zealand palaeoclimate from the last interglacial to the global last glacial maximum. Quat. Sci. Rev. 110, 92–106. (doi:10.1016/j.quascirev.2014.12.017)

[39] Hewitt GM 1996 Some genetic consequences of ice ages, and their role in divergence and speciation. Biol. J. Linn. Soc. 58, 247–276. (doi:10.1111/j.1095-8312.1996.tb01434.x)

[40] Taberlet P, Fumagalli L, Wust-Saucy A-G, Cossons J-F 1998 Comparative phylogeography and postglacial colonization. Mol. Ecol. 7, 453–464. (doi:10.1046/j.1365-294x.1998.00289.x) 9628000 10.1046/j.1365-294x.1998.00289.x

[41] Avise JC 2000 Phylogeography: the history and formation of species, 449p Cambridge, UK: Harvard University Press.

[42] Trewick S, Bland K 2012 Fire and slice: palaeogeography for biogeography at New Zealand's North Island/South Island juncture. J. R. Soc. N. Z. 42, 153–183. (doi:10.1080/03036758.2010.549493)

[43] Shepherd LD, Perrie LR, Brownsey PJ 2007 Fire and ice: volcanic and glacial impacts on the phylogeography of the New Zealand forest fern *Asplenium hookerianum*. Mol. Ecol. 16, 4536–4549. (doi:10.1111/j.1365-294X.2007.03451.x) 17877716 10.1111/j.1365-294X.2007.03451.x

[44] Bulgarella M, Trewick SA, Minards NA, Jacobson MJ, Morgan-Richards M 2014 Shifting ranges of two tree weta species (*Hemideina* spp.): competitive exclusion and changing climate. J. Biogeogr. 41, 524–535. (doi:10.1111/jbi.12224)

[45] Morgan-Richards M 2000 Robertsonian translocations and B chromosomes in the Wellington tree weta, *Hemideina crassidens* (Orthoptera: Anostostomatidae). Hereditas 132, 49–54. (doi:10.1111/j.1601-5223.2000.00049.x) 10857259 10.1111/j.1601-5223.2000.00049.x

[46] Bridgeman B 2016 *Detection of the bacterial endosymbiot* Wolbachia *and determination of super groups present within New Zealand invertebrates* Palmerston North, New Zealand: Massey University.

[47] Miller MP 2005 Alleles In Space (AIS): Computer software for the joint analysis of interindividual spatial and genetic information. J. Hered. 96, 722–724. (doi:10.1093/jhered/esi119) 16251514 10.1093/jhered/esi119

[48] Miller MP, Haig SM, Wagner RS 2006 Phylogeography and spatial genetic structure of the Southern torrent salamander: implications for conservation and management. J. Hered. 97, 561–570. (doi:10.1093/jhered/esl038) 17135462 10.1093/jhered/esl038

[49] Miller MP, Bellinger MR, Forsman ED, Haig SM 2006 Effects of historical climate change, habitat connectivity, and vicariance on genetic structure and diversity across the range of the red tree vole (*Phenacomys longicaudus*) in the Pacific Northwestern United States. Mol. Ecol. 15, 145–159. (doi:10.1111/j.1365-294X.2005.02765.x) 16367837 10.1111/j.1365-294X.2005.02765.x

[50] Funk WC, Forsman ED, Mullins TD, Haig SM 2008 Landscape features shape genetic structure in threatened northern spotted owls. Open file report, US Geological Survey, Reston, VA, USA, pp. 1–11. 10.1111/j.1752-4571.2007.00002.x PMC3352401 25567499

[51] Mckean NE, Trewick SA, Morgan-Richards M 2016 Little or no gene flow despite F1 hybrids at two interspecific contact zones. Ecol. Evol. 6, 2390–2404. (doi:10.1002/ece3.1942) 27066230 10.1002/ece3.1942 PMC4783458

[52] Field LH, Rind FC 1992 Stridulatory behaviour in a New Zealand weta, *Hemideina crassidens*. J. Zool. 228, 371–394. (doi:10.1111/j.1469-7998.1992.tb04442.x)

[53] Field LH, Bigelow RS 2001 Morphometric analysis of *Hemideina* spp. in New Zealand. In The biology of wetas, king crickets and their allies (ed. Field LH), pp. 163–177. Guildford, UK: CABI Publishing.

[54] Kearse M et al. 2012 Geneious Basic: an integrated and extendable desktop software platform for the organization and analysis of sequence data. Bioinformatics 28, 1647–1649. (doi:10.1093/bioinformatics/bts199) 22543367 10.1093/bioinformatics/bts199 PMC3371832

[55] Tajima F 1993 Measurement of DNA polymorphism. In Mechanism of molecular evolution, introduction to molecular paleopopulation biology. (eds Takahata N, Clark AG), pp. 37–59. Sunderland, MA: Japan Scientific Societies Press, Sinauer Associates Inc.

[56] Haag-Liautard C, Coffey N, Houle D, Lynch M, Charlesworth B, Keightley PD 2008 Direct estimation of the mitochondrial DNA mutation rate in *Drosophila melanogaster*. PLoS Biol. 6, 1706–1714. (doi:10.1371/journal.pbio.0060204) 10.1371/journal.pbio.0060204 PMC2517619 18715119

[57] Xu S, Schaack S, Seyfert A, Choi E, Lynch M, Cristescu ME 2012 High mutation rates in the mitochondrial genomes of *Daphnia pulex*. Mol. Biol. Evol. 29, 763–769. (doi:10.1093/molbev/msr243) 21998274 10.1093/molbev/msr243 PMC3350313

[58] Molnar RI, Bartelmes G, Dinkelacker I, Witte H, Sommer RJ 2011 Mutation rates and intraspecific divergence of the mitochondrial genome of *Pristionchus pacificus*. Mol. Biol. Evol. 28, 2317–2326. (doi:10.1093/molbev/msr057) 21368317 10.1093/molbev/msr057

[59] Bandelt HJ, Forster P, Rohl A 1999 Median-joining networks for inferring intraspecific phylogenies. Mol. Biol. Evol. 16, 37–48. (doi:10.1093/oxfordjournals.molbev.a026036) 10331250 10.1093/oxfordjournals.molbev.a026036

[60] Leigh JW, Bryant D 2015 POPART: Full-feature software for haplotype network construction. Methods Ecol. Evol. 6, 1110–1116. (doi:10.1111/2041-210X.12410)

[61] Morgan-Richards M, Bulgarella M, Sivyer L, Dowle EJ, Hale M, McKean NE, Trewick SA 2017 Data from: Explaining large mitochondrial sequence differences within a population sample Dryad Digital Repository. (http://dx.doi.org/10.5061/dryad.rg15p) 10.1098/rsos.170730 PMC5717637 29291063

[62] Twort VG, Dennis AB, Park D, Lomas KF, Newcomb RD, Buckley TR 2017 Positive selection and comparative molecular evolution of reproductive proteins from New Zealand tree weta (Orthoptera, *Hemideina*). PLoS ONE 12, e0188147 (doi:10.1371/journal.pone.0188147) 29131842 10.1371/journal.pone.0188147 PMC5683631

[63] King TM, Hanotte O, Burke T, Wallis GP 1997 Characterization of four microsatellite loci in tree weta (Orthoptera: Stenopelmatidae): their potential usefulness for the study of *Hemideina*. Mol. Ecol. 7, 657–666.

[64] Hale ML, Alabergère G, Hale RJ 2010 Polymorphic microsatellite loci for the Banks Peninsula tree weta *Hemideina ricta*, and cross amplification in *H. femorata*. Conserv. Genet. Resour. 2(Suppl.1), 329–331. (doi:10.1007/s12686-010-9232-3)

[65] Raymond M, Rousset F 1995 An exact test for population differentiation. Evolution 49, 1280–1283. 28568523 10.1111/j.1558-5646.1995.tb04456.x

[66] Rousset F 2008 GENEPOP'007: a complete re-implementation of the GENEPOP software for Windows and Linux. Mol. Ecol. Resour. 8, 103–106. (doi:10.1111/j.1471-8286.2007.01931.x) 21585727 10.1111/j.1471-8286.2007.01931.x

[67] Zhang Z, Li J, Zhao XQ, Wang J, Wong GK, Yu J 2006 KaKs_calculator: calculating Ka and Ks through model selection and model averaging. Genomics Proteomics Bioinformatics 4, 259–263. (doi:10.1016/S1672-0229(07)60007-2) 17531802 10.1016/S1672-0229(07)60007-2 PMC5054075

[68] Dowle EJ 2013 Rates of molecular evolution and gene flow. Palmerston North, New Zealand: Massey University.

[69] Martin M 2011 Cutadapt removes adapter sequences from high-throughput sequencing reads. EMBnet.journal. 17, 10 (doi:10.14806/ej.17.1.200)

[70] Cox MP, Peterson DA, Biggs PJ 2010 SolexaQA: at-a-glance quality assessment of Illumina second-generation sequencing data. BMC Bioinformatics 11, 485 (doi:10.1186/1471-2105-11-485) 20875133 10.1186/1471-2105-11-485 PMC2956736

[71] Zerbino DR, Birney E 2008 Velvet: algorithms for de novo short read assembly using de Bruijn graphs. Genome Res. 18, 821–829. (doi:10.1101/gr.074492.107) 18349386 10.1101/gr.074492.107 PMC2336801

[72] Simpson JT, Wong K, Jackman SD, Schein JE, Jones SJM, Birol I 2009 ABySS: a parallel assembler for short read sequence data. Genome Res. 19, 1117–1123. (doi:10.1101/gr.089532.108) 19251739 10.1101/gr.089532.108 PMC2694472

[73] Kim I, So YC, Myung HY, Jae SH, Sang ML, Hung DS, Byung RJ 2005 The complete nucleotide sequence and gene organization of the mitochondrial genome of the oriental mole cricket, *Gryllotalpa orientalis* (Orthoptera: Gryllotalpidae). Gene 353, 155–168. (doi:10.1016/j.gene.2005.04.019) 15950403 10.1016/j.gene.2005.04.019

[74] Odong TL, van Heerwaarden J, van Hintum TJL, van Eeuwijk FA, Jansen J 2013 Improving hierarchical clustering of genotypic data via principal component analysis. Crop Sci. 53, 1546–1554. (doi:10.2135/cropsci2012.04.0215)

[75] Fraley C, Raftery AE 1999 MCLUST: software for model-based cluster analysis. J. Classif. 16, 297–306. (doi:10.1007/s003579900058)

[76] Fraley C, Raftery AE, Murphy B, Scrucca L 2012 Mclust version 4 for R: normal mixture modeling for model-based clustering, classification, and density estimation. Technical report 597, University of Washington, Seattle, WA, USA.

[77] Nanova O 2014 Geographical variation in the cranial measurements of the midday jird *Meriones meridianus* (Rodentia: Muridae) and its taxonomic implications. J. Zool. Syst. Evol. Res. 52, 75–85. (doi:10.1111/jzs.12032)

[78] Charlesworth D 2006 Balancing selection and its effects on sequences in nearby genome regions. PLoS Genet. 2, 379–384. (doi:10.1371/journal.pgen.0020064) 10.1371/journal.pgen.0020064 PMC1449905 16683038

[79] Hedrick PW 2007 Balancing selection. Curr. Biol. 17, 230–231. (doi:10.1016/j.cub.2007.01.012) 17407748 10.1016/j.cub.2007.01.012

